# The Overlapping Genetics of Amyotrophic Lateral Sclerosis and Frontotemporal Dementia

**DOI:** 10.3389/fnins.2020.00042

**Published:** 2020-02-05

**Authors:** Yevgeniya A. Abramzon, Pietro Fratta, Bryan J. Traynor, Ruth Chia

**Affiliations:** ^1^Neuromuscular Diseases Research Section, Laboratory of Neurogenetics, National Institute on Aging, NIH, Bethesda, MD, United States; ^2^Sobell Department of Motor Neuroscience and Movement Disorders, Institute of Neurology, University College London, London, United Kingdom; ^3^Department of Neurology, Brain Science Institute, Johns Hopkins University, Baltimore, MD, United States

**Keywords:** amyotrophic lateral sclerosis, frontotemporal dementia, neurological disorders, neurodegeneration, overlapping genetics

## Abstract

Amyotrophic lateral sclerosis (ALS) and frontotemporal dementia (FTD) are two diseases that form a broad neurodegenerative continuum. Considerable effort has been made to unravel the genetics of these disorders, and, based on this work, it is now clear that ALS and FTD have a significant genetic overlap. TARDBP, SQSTM1, VCP, FUS, TBK1, CHCHD10, and most importantly C9orf72, are the critical genetic players in these neurological disorders. Discoveries of these genes have implicated autophagy, RNA regulation, and vesicle and inclusion formation as the central pathways involved in neurodegeneration. Here we provide a summary of the significant genes identified in these two intrinsically linked neurodegenerative diseases and highlight the genetic and pathological overlaps.

## Introduction

Amyotrophic lateral sclerosis (ALS, OMIM #105400) is a fatal neurological disorder affecting motor neurons located in the frontal cortex, brainstem, and spinal cord (Cleveland and Rothstein, 2001). The disease typically begins as muscle weakness in a limb, or occasionally with changes in voice or difficulty swallowing, which progresses to generalized weakness and paralysis of respiratory muscles leading to death due to respiratory failure. Approximately 10% of all ALS cases have a family history of the disease, while the remaining 90% are sporadic. The incidence of ALS is estimated to be 2.1 new cases per 100,000 population per year (Chio et al., 2013), and approximately 6,000 people are newly diagnosed with ALS each year in the United States alone. The number of ALS cases around the world is increasing due to the aging of the global population (Arthur et al., 2016). There are currently no effective treatments for ALS, except for edaravone, which reduces the decline in daily functioning, and riluzole, which prolongs patients’ survival by a few months (Miller et al., 2012; Rothstein, 2017).

Frontotemporal degeneration (FTD) is one of the most common types of dementia in people under 65. FTD may be divided into three primary subtypes, namely behavioral variant, semantic dementia, and progressive non-fluent aphasia. Among these subtypes, behavioral variant FTD is the most commonly observed type of dementia associated with motor neuron disorders (Bird et al., 1999). The incidence of FTD is approximately 4.0 new cases per 100,000 population per year, with 40% of cases being familial (Ratnavalli et al., 2002). Similar to ALS and other neurological disorders, there is no effective treatment for FTD (Tsai and Boxer, 2014).

It is now recognized that ALS and FTD are two diseases that form a broad neurodegenerative continuum. One of the earliest hints of this overlap came from the clinical observation that both disorders can be present within the same family or even within the same individual. Cross-sectional studies performed over the last decade estimate that up to 50% of ALS patients develop cognitive impairment associated with FTD. Similarly, up to 30% of FTD patients develop motor dysfunction (Burrell et al., 2011).

Considerable progress has been made in unraveling the genetics of ALS and FTD, and it is now clear that the genetics of these two neurodegenerative conditions overlap significantly. TARDBP, SQSTM1, VCP, FUS, TBK1, CHCHD10, and most importantly C9orf72, are the critical genetic players, and their discoveries have implicated autophagy, RNA processing, and vesicle and inclusion formation as the central pathways involved in these forms of neurodegeneration.

Here we provide a summary of the significant genes identified in these two intrinsically linked neurodegenerative diseases and highlight where cross-talk exists. We describe the genes in the order of their relevance to ALS/FTD overlap, ranging from genes that have been demonstrated to cause both clinically and neuropathologically confirmed ALS and FTD to genes where the cognitive or motor symptoms are reported in the literature but pathological confirmation in not yet available.

The genes described in this review, clinical phenotypes and pathways associated with them are summarized in Table 1.

**TABLE 1:** Key genes identified in ALS and FTD.

**Gene**	**Locus**	**Neurological phenotypes**	**Pathway**	**Main localization**
FUS	16p11.2	ALS, FTD, ALS (juvenile with BIs) ET, MND (lower), bvFTD?, PD?	Nucleocytoplasmic transport/splicing	Nucleus
TDP-43	1p36.22	ALS, FTD, ALS (flail arm variant), SNGP and chorea, MND	Nucleocytoplasmic transport/splicing	Nucleus
CHCHD10	22q11.23	ALS, ALS/FTD, Mitochondrial myopathy (autosomal dominant)	Mitochondrial dysfunction/Synaptic integrity	Mitochondrion, nucleus
C9orf72	9p21.2	AD, ALS, FTD, ALS/FTD, BD, PD, Schizophrenia	Nucleocytoplasmic transport/splicing	Extracellular, nucleus, endosome, lysosome
UBQLN2	Xp11.21	ALS, FTD, Neurodegeneration, X-linked	Autophagy/Proteasome	Cytosol, plasma membrane, nucleus
TBK1	12q14.1	ALS, ALS/FTD, AD	Autophagy/inflammation	Nucleus, cytosol, endosome, mitochondrion
VCP	9p13.3	IBMPFD, ALS, IBMPFD and ALS, CMT2, HSP DMRV, Scapuloperoneal muscular dystrophy and dropped head fibers, AD?, Autism?	Autophagy/Mitochondrial function	Nucleus, endoplasmic reticulum, cytosol, extracellular, lysosome
SQSTM1	5q35	PDB, ALS, FTD, AD, early onset ALS/FTD, NADGP	Autophagy	Nucleus, cytosol, lysosome, endoplasmic reticulum, endosome

*IBMPFD, inclusion body myopathy with Paget disease and frontotemporal dementia; AD, Alzheimer disease; ALS, amyotrophic lateral sclerosis; FTD, frontotemporal dementia; HSP, hereditary spastic paraplegia; CMT2, charcot-marie-tooth disease, type 2; PDB, paget’s disease of bone; PD, Parkinson disease; BD, bipolar disorder; MND, motor neuron disease; ET, essential tremor; Bis, basophilic inclusions; DMRV-Myopathy, rimmed vacuolar; NADGP, neurodegeneration, childhood onset with ataxia, dystonia and gaze palsy; SNGP, supranuclear gaze palsy.*

## Chromosome 9 Open Reading Frame 72 (C9Orf72)

In 2011, a hexanucleotide repeat expansion within the C9orf72 gene located on chromosome 9p21 was identified as a significant genetic cause of both ALS and FTD (DeJesus-Hernandez et al., 2011; Renton et al., 2011). This repeat expansion is the most common genetic cause of ALS, FTD, and ALS/FTD responsible for ∼11% of all ALS and ∼13% of all FTD cases. This discovery demonstrated that there is a more considerable genetic overlap between ALS and FTD than had been previously estimated. The majority of C9orf72-related FTD cases manifest behavioral symptoms with a much smaller percentage presenting with semantic dementia or with progressive non-fluent aphasia. C9orf72 repeat expansions have also been implicated as rare causes of other neurodegenerative diseases, including Alzheimer’s disease, Parkinson’s disease, progressive supranuclear palsy, ataxia, corticobasal syndrome, Huntington disease-like syndrome, and Creutzfeldt–Jakob disease (Beck et al., 2013; Hensman Moss et al., 2014; Devenney et al., 2018).

Several mechanisms have been proposed to explain how C9orf72 expansion causes neurological disease. These include (Cleveland and Rothstein, 2001) haploinsufficiency of C9orf72 protein (DeJesus-Hernandez et al., 2011; Renton et al., 2011; Chio et al., 2013; Shi et al., 2018; Shao et al., 2019) RNA toxicity due to accumulation of RNA containing the GGGGCC repeat in the brain and spinal cord (DeJesus-Hernandez et al., 2011; Renton et al., 2011; Arthur et al., 2016; Arzberger et al., 2018) dipeptide repeat (DPR) protein toxicity arising from repeat-associated non-AUG translation occurring off the expansion (Miller et al., 2012; May et al., 2014; Freibaum and Taylor, 2017) disruption of the nucleocytoplasmic transport (Freibaum et al., 2015; Jovicic et al., 2015; Zhang et al., 2015). Although the data for each of these mechanisms are compelling, it is not yet clear which of them plays the dominant role in determining neurodegeneration. The possibility of multiple mechanisms, operating in either unison or sequentially to bring about neuronal death, cannot be discounted.

Various mouse models have been created to elucidate the pathogenic mechanism underlying C9orf72 neurodegeneration. Though informative, these models have failed to resolve the exact mechanism, as the available information bolsters all four modes of neurodegeneration. For example, mice lacking C9orf72 in neurons and glial cells did not display motor neuron degeneration or defects in motor function associated with ALS (Koppers et al., 2015). BAC transgenic mice with expanded human C9orf72 hexanucleotide repeat that ranged between 100 and 1000 repeats developed RNA foci and dipeptide repeat proteins throughout the nervous system. However, there was no evidence of neurodegeneration or functional deficits (O’Rourke et al., 2015; Peters et al., 2015). Mice with more than 450 GGGGCC repeats have mild hippocampal neuronal loss and display signs of age-dependent anxiety and impaired cognitive functioning (Jiang et al., 2016).

More recent mouse models showed that that loss of C9orf72 in a gain-of-function C9ALS/FTD mouse model aggravates motor behavior deficits in a dose-dependent manner (Shao et al., 2019). Transgenic GFP-PR28 mice expressing arginine-rich poly(PR), the most toxic type of DPRs in neurons, did partially develop neuropathological features of C9FTD/ALS (Hao et al., 2019). Two other transgenic C9FTD/ALS mouse models demonstrated that poly(GR) affects translation and stress granule dynamics (Zhang et al., 2018) and compromises mitochondrial function by binding Atp5a1 (Choi et al., 2019).

## TAR DNA-Binding Protein 43 (TARDBP)

Mutations in the TAR DNA-binding protein 43 (the gene that encodes the TDP-43 protein) were linked to ALS in 2008 (Sreedharan et al., 2008). Before that, it was recognized that TDP-43 cytoplasmic and nuclear inclusions are characteristic of both ALS and FTD. In ALS, the cytoplasmic accumulation of TDP-43 is found in neurons and glia of the primary motor cortex, brainstem motor nuclei, and spinal cord (Bodansky et al., 2010; Mackenzie et al., 2010). In FTD, the inclusions are observed in the neocortex and dentate granule cells of the hippocampus (Neumann et al., 2006; Davidson et al., 2007). TDP-43 mutations are the cause of ∼ 1% of all ALS cases. In contrast, an even smaller number of FTD cases arising from mutations in this gene have been described, despite the widespread presence of TDP-43 in FTD brains (Tan et al., 2017).

TDP-43 is a DNA and RNA binding protein involved in many aspects of RNA metabolism, including splicing, microRNA biogenesis, transcription, and stabilization of messenger RNA (Buratti et al., 2001; Strong et al., 2007; Buratti and Baralle, 2008; Fiesel et al., 2010; Lagier-Tourenne et al., 2010). Two contrasting mechanisms have been proposed to explain TDP-43 related neurodegeneration, namely (Cleveland and Rothstein, 2001) loss of function arising from sequestration of critical TDP-43 protein within cytoplasmic aggregates leading to nuclear depletion of TDP-43 (Chio et al., 2013; Mitra et al., 2019; Roczniak-Ferguson and Ferguson, 2019) gain of function effect due to some inherent toxic property of the aggregates (Buratti and Baralle, 2012; Hergesheimer et al., 2019). However, the toxic role of aggregated TDP-43 in neurodegeneration is still under debate. Recent research has focused on the role of stress granules in the pathogenesis of TDP43-related ALS (Khalfallah et al., 2018). TDP-43 mutations have also been reported to alter liquid drop formation, though the pathophysiological role of this *in vitro* epiphenomena remains unclear (Conicella et al., 2016).

More than fifteen mouse models have been created in the last 2 years in an attempt to decipher the pathogenic roles of TDP-43 in autophagy, protein homeostasis, and clearance pathways involved in ALS and FTD. These rodent models showed that suppression of conditional TDP-43 transgene expression differentially affects early cognitive and social phenotypes in TDP-43 mice (Silva et al., 2019). In a TDP-43Q331K/Q331K knock-in mouse model of ALS-FTD, TDP-43 gains function due to impaired autoregulation (White et al., 2018b). In TDP-43M337V and TDP-43G298S knock-in mice, mutant TDP-43 causes early stage dose-dependent motor neuron degeneration (Ebstein et al., 2019). Mice with endogenous TDP-43 mutations exhibit gain of splicing function and characteristics of motor neuron degeneration (Fratta et al., 2018). Mouse models have also provided insight into how mutations in this gene may be underlying frontotemporal dementia. A recent TDP-43Q331K mouse model manifested cognitive dysfunction in the absence of motor dysfunction. Pathological examination showed that normal localization of TDP-43 within the cell, but there was evidence of perturbed regulation of TDP-43 (White et al., 2018a, b).

## Sequestosome-1 (SQSTM1)

Mutations in Sequestosome-1 (SQSTM1) was initially discovered in patients with Paget’s disease of bone (Laurin et al., 2002) and linked to ALS and behavioral FTD in 2011 (Fecto et al., 2011). SQSTM1 encodes p62, a multifunctional protein involved in a wide range of cellular functions, including apoptosis (Jung and Oh, 2019), NFKB1 signaling (Foster et al., 2019), ubiquitin-mediated autophagy (Zaffagnini et al., 2018; Gao et al., 2019; Park et al., 2019), and transcription regulation (Rea et al., 2013). p62 is also a standard component of ubiquitin-containing inclusions in several neurological disorders, including ALS and FTD. More than 100 variants have been identified in SQSTM1, and cumulatively they account for ∼1% of all ALS and up to 3% of all FTD cases. Defective p62 is prone to forming aggregates. Individuals with SQSTM1 variants have p62-positive inclusions in the motor neurons if presenting with ALS, and in the hippocampus and cerebral neocortex if presenting with FTD (Arai et al., 2003; Teyssou et al., 2013).

Accumulation of SQSTM1 comes from disturbances in the selective autophagy pathway (Deng et al., 2019). However, the pathogenic mechanism that contributes to SQSTM1-related impaired autophagy and degradation remains poorly understood. Similar to TDP-43 and FUS, SQSTM1 goes through liquid-liquid phase separation. Recent research shows that cytoplasmic DAXX drives SQSTM1/p62 phase condensation, an essential step in the activation of Nrf2-mediated stress response (Yang et al., 2019). Polyubiquitin chain-induced p62 phase separation leads to the segregation of autophagic cargo (Herhaus and Dikic, 2018; Sun et al., 2018).

To date, no. p62 mouse model has been created to study the direct effect of p62 mutations in ALS/FTD. However, many mouse models exist that demonstrate a relationship between p62 and other ALS genes. Mitsui et al. (2018) previously reported that loss of SQSTM1 exacerbates disease phenotypes in SOD1H46R ALS mice. Following the initial report, the same authors demonstrated that SQSTM1 overexpression results in a significant increase in biochemically detectable insoluble SQSTM1 and poly-ubiquitinated proteins in the spinal cord of SQSTM1; SOD1H46R mice when compared to SOD1H46R mice. This observation suggests that overexpression of p62 in SOD1H46R mice accelerates disease onset by impairing the protein degradation pathways (Mitsui et al., 2018).

From the FTD perspective, apart from developing mature-onset obesity due to impaired glucose tolerance and insulin resistance, p62 knockout mice display significantly reduced life span and accelerated aging phenotypes. These mice develop cognitive impairment and anxiety, which are symptoms characteristic of human Alzheimer’s disease (Kwon et al., 2012).

## Fused in Sarcoma (FUS)

Fused in sarcoma (FUS) is an RNA-binding protein that was linked to ALS in 2009 (Kwiatkowski et al., 2009). Similar to TDP-43, FUS is involved in multiple aspects of RNA metabolism regulation, including alternative splicing, RNA translation, and transport (Kwiatkowski et al., 2009; Vance et al., 2009). Mutations in FUS are responsible for ∼1% of all ALS. They are also occasionally observed in behavioral FTD cases. In addition to these phenotypes, abnormal aggregates of FUS, independently of their mutations, are present in other neurodegenerative diseases such as hereditary essential tremor, the polyglutamine diseases, and Parkinson’s disease.

Amyotrophic lateral sclerosis and FTD related mutations are clustered in highly conserved regions of the gene and affect the protein nuclear localization signal (NLS). Similar to TDP-43, mutations in the FUS gene are predominantly found in ALS patients. A limited number of FUS mutations (p.P106L, p.Gly174-Gly175 deletion GG, p.M254V) have been described in FTD patients without concomitant ALS (Van Langenhove et al., 2010; Huey et al., 2012).

Two mechanisms were proposed to explain FUS-related neurodegeneration. First of all, there is the toxic gain-of-function in which nuclear FUS aggregates in cytoplasm and spreads in a prion-like manner through neuronal tissues (Armstrong, 2017). Second, the depletion of FUS from the nucleus may impair transcription, alternative splicing, and DNA repair (Shang and Huang, 2016). A reasonable amount of evidence supports both mechanisms, and different mechanisms may stand behind different FUS mutations (Ishigaki and Sobue, 2018; An et al., 2019). Liquid–liquid phase separation (LLPS) of FUS has emerged recently as an alternative mechanism for FUS-related neurodegeneration. It is now established that LLPS is modulated by universal cellular actors such as ATP and nucleic acids through enhancement and dissolution (Kang et al., 2019). Other recent FUS studies expanded on LLPS functions, mechanism, and transformation (Berry et al., 2018; Kang et al., 2019; Murthy et al., 2019; Niaki et al., 2019).

Multiple mouse models have been created in an attempt to identify the pathogenic roles of FUS in neurodegeneration. FUS knockout mice display behavioral abnormalities such as hyperactivity and reduced anxiety-related behavior. However, they do not develop motor neuron impairment, suggesting that the ablation of the FUS gene alone is insufficient to cause ALS (Kino et al., 2015). Transgenic mice overexpressing exogenous FUS with nuclear localization signal deletion (ΔNLS-FUS) under Thy1 neuron-specific promoter develop progressive ALS phenotypes associated with the formation of ubiquitin/p62-positive FUS aggregates, neuronal loss, and gliosis. In FusΔNLS/ΔNLS mice, truncation of the NLS region leads to mislocalization of FUS protein from the nucleus to the cytoplasm in spinal motor neurons and cortical neurons where it leads to apoptosis (Scekic-Zahirovic et al., 2016). Furthermore, both FusΔNLS/+mice and knock-in mice carrying another C-terminal frameshift mutation (FusΔ14/+) develop progressive motor neuron loss in heterozygosity, recapitulating the early stages of disease (Scekic-Zahirovic et al., 2016; Devoy et al., 2017). More recent FUSR514G and FUSR521C transgenic mice models show that overriding the FUS autoregulation system triggers gain-of-function toxicity via an altered autophagy-lysosome pathway and impaired RNA metabolism (Ho and Ling, 2019; Ling et al., 2019).

## Valosin Containing Protein (VCP)

Mutations in Valosin containing protein (VCP) was initially discovered as the cause of a clinical syndrome characterized by the triad of inclusion body myopathy, Paget’s disease of bone, and frontotemporal dementia (IBMFTD) in 2004 (Watts et al., 2004). Mutations in this gene were subsequently identified as a cause of ALS, representing an early example of how genetic mutations in a single gene could underlie both ALS and FTD (Johnson et al., 2010). To date, 72 autosomal dominant mutations have been discovered in this gene, more than 30 of which are reported in ALS or FTD cases (including behavioral FTD, semantic dementia, and progressive non-fluent aphasia) (Al-Obeidi et al., 2018; Saracino et al., 2018; Bastola et al., 2019). Many of the reported VCP mutations are located on exon five within the N-terminal CDC48 domain, which is involved in ubiquitin-binding, meaning that mutations in this region may negatively affect the ubiquitin protein degradation pathway (Ganji et al., 2018; Twomey et al., 2019).

A recent study by Al-Obeidi et al. (2018) showed that VCP mutations are present in ∼9% of ALS, 4% of Parkinson’s disease, and 2% of Alzheimer’s disease patients. As of today, no definite correlation between the mutation type and the incidence of clinical features associated with VCP has been established (Al-Obeidi et al., 2018; Plewa et al., 2018).

Valosin Containing Protein encodes a member of the AAA-ATPase enzyme family with wide-ranging functions in cell division (Ogura and Wilkinson, 2001), DNA repair, ubiquitin-dependent protein degradation, and suppression of apoptosis (Ogura and Wilkinson, 2001). Ludtmann et al. (2017) provides evidence that mutations in VCP lead to mitochondrial uncoupling due to a reduced ADP/ATP translocation by adenine nucleotide translocase. Such deficiency in mitochondrial bioenergetics makes neurons especially vulnerable as they require more energy than other cell types (Ludtmann et al., 2017).

Recent mouse models of VCP showed that activation of the NLRP3 inflammasome is associated with VCP protein myopathy. Nalbandian et al. (2017) reported a significant increase in the expression of NLRP3, Caspase 1, IL-1β, and IL-18 in the quadriceps of 12 and 24 months old VCPR155H/+heterozygous mice. Furthermore, a significant increase of IL-1β(+)F4/80(+)Ly6C(+) macrophages in the quadriceps and bones of the same mice were also observed and is positively correlated with high expression levels of TDP-43 and p62/SQSTM1 markers of VCP pathology and progressive muscle wasting (Nalbandian et al., 2017).

Another recent discovery showed that VCP plays a vital role in the maintenance of lysosomal homeostasis and TFEB activity in differentiated skeletal muscle (Arhzaouy et al., 2019). Arhzaouy et al. (2019) showed that selective inactivation of VCP in skeletal muscles of Myl1p-cre-vcp-/-mice, results in a necrotic myopathy with increased macroautophagic/autophagic proteins and damaged lysosomes. It was further demonstrated that the myofiber necrosis was preceded by the upregulation of LGALS3/Galectin-3, a marker of damaged lysosomes, and TFEB activation, suggesting early defects in the lysosomal system (Arhzaouy et al., 2019).

## Coiled-Coil-Helix-Coiled-Coil-Helix Domain Containing 10 (CHCHD10)

Coiled-coil-helix-coiled-coil-helix domain-containing protein 10 (CHCHD10) is a mitochondrial protein associated with ALS and FTD, including the behavioral and primary progressive aphasia subtypes of this form of dementia (Ajroud-Driss et al., 2015; Cozzolino et al., 2015). The protein was discovered in 2014 by exome sequencing of a large French family affected by autosomal dominant FTD with or without ALS, cerebellar ataxia, and mitochondrial myopathy (Chaussenot et al., 2014). At least 30 variants have since been reported, and they are concentrated on exon two of the gene encoding the non-structured N-terminal (Taylor et al., 2016; Perrone et al., 2017; Zhou et al., 2017).

Coiled-Coil-Helix-Coiled-Coil-Helix Domain Containing 10 is a multifunctional protein involved in the regulation of mitochondrial metabolism, synthesis of respiratory chain components, and modulation of cell apoptosis (Zhou et al., 2017). Perhaps not surprisingly, mutations in CHCHD10 lead to disassembly of the mitochondrial contact site complex, severe mitochondrial DNA repair deficiency after oxidative stress, disruption of oxygen consumption and ATP synthesis in cells, and disturbance of apoptotic mechanisms (Zhou et al., 2017).

Recent data shows enrichment of CHCHD10 expression at the postsynaptic membrane of neuromuscular junctions (Bannwarth et al., 2014; Zhou et al., 2017; Xiao et al., 2019). Deletion of CHCHD10 in skeletal muscle of HSA-CHCHD10-/- knockout mice results in motor defects and neurotransmission impairment, indicating that muscle CHCHD10 is required for normal neurotransmission between motoneurons and skeletal muscle fibers (Xiao et al., 2019). Furthermore, an examination of HSA-CHCHD10-/- mice mitochondria under an electron microscope revealed a large quantity of large lysosome-like vesicles, indicating active mitochondria degradation and suggesting that CHCHD10 is required for mitochondria structure and ATP production (Burstein et al., 2018; Xiao et al., 2019).

Two groups independently developed CHCHD10S55L knock-in mice, representative of human CHCHD10 S59L mutation, and found that these mice developed progressive motor deficits, myopathy, cardiomyopathy, and died prematurely (Anderson et al., 2019; Genin et al., 2019). Histological examination revealed that CHCHD10, together with its twin CHCHD2 forms aggregates resulting in abnormal organelle morphology and function. In contrast, knock out CHCHD10 mice containing a single adenine nucleotide insertion in exon two that results in a prematurely terminated protein, did not develop similar pathology, suggesting that tissue-specific toxic gain-of-function is the likely mechanism behind CHCHD10 S59L related neurodegeneration (Anderson et al., 2019).

## TANK-Binding Kinase 1 (TBK1)

TANK-binding kinase 1 (TBK1) gene was discovered in 2015 through the whole-exome sequencing analysis of a large case-control cohort (Cirulli et al., 2015; Freischmidt et al., 2015). In 2016, a large genome-wide association study (GWAS) also identified the TBK1 gene on chromosome 12q14.2 as a risk locus for ALS, thus confirming the gene’s association with motor neuron degeneration (van Rheenen et al., 2016). TBK1 is a member of the IkB kinase family involved in autophagy, mitophagy, and innate immune signaling (Weidberg and Elazar, 2011). The protein is highly expressed in neuronal cells of the cerebral cortex, hippocampus, and lateral ventricle (Uhlen et al., 2015). It also interacts with other genes implicated in ALS, such as OPTN and SQSTM1, to form TBK1 autophagic adaptor complex (Ryzhakov and Randow, 2007; Morton et al., 2008; Li et al., 2016).

To date, more than 90 mutations have been discovered on TBK1. According to a recent meta-analysis study, TBK1 loss of function and missense mutations account for 1.0 and 1.8% in ALS/FTD patients, respectively (Lamb et al., 2019). The majority of TBK1 mutations are loss of function that result in the deletion of the C-terminal domain responsible for interaction with adaptor proteins that regulate the cellular distribution of TBK1 and activation of downstream signaling pathways (Ryzhakov and Randow, 2007). Indeed, mutations appear to lead to a significant decrease in TBK1 expression at the mRNA and protein levels (Freischmidt et al., 2015).

TANK-binding kinase 1 mutations are associated with bulbar onset ALS and fast progressing behavioral FTD (Freischmidt et al., 2015). In ALS patients, TBK1 mutations are pathologically characterized by TDP-43 positive and p62 positive inclusions in motor neurons, as well as TDP-43 inclusions in the cortex. Similar to that observed in ALS, FTD patients, harboring TBK1 mutations is also characterized by TDP-43 inclusions in numerous brain regions and cytoplasmic p62 and ubiquitin-positive inclusions in glial cells (Van Mossevelde et al., 2016).

Compelling evidence exists that loss-of-function is the pathological mechanism behind TBK1-related ALS and FTD (de Majo et al., 2018; Lamb et al., 2019; Weinreich et al., 2019). Germline deletion of TBK1 is lethal in embryonic mice suggesting that the protein plays a critical role in developmental homeostasis (Bonnard et al., 2000). More recent rodent models demonstrated that conditional neuron-specific knockout of Tbk1 in Tbk1fl/fl Nestin-Cre mice leads to the development of cognitive and motor dysfunction similar to ALS/FTD. Neuron-specific Tbk1 deletion induces morphological and biochemical alterations in neurons and glia such as abnormal dendrites, neurofibrillary tangles, reduced dendritic spine density, as well as cortical synapse loss. Furthermore, Tbk1 knockout impairs autophagy in motor neuron-like cells, while Tbk1 over-expression extends survival of ALS transgenic mice (Duan et al., 2019).

TANK-Binding Kinase 1 is a central regulator of selective autophagy and inflammatory responses via IFN type I signaling (Perry et al., 2004; Hu et al., 2018). Heterozygous deletion of the α-IFN receptor Ifnar1 significantly prolongs the life span of SOD1G93A ALS mice (Wang et al., 2011). In a 2019 study, Brenner et al. (2019) further elucidated on the connection between TBK1 and SOD1 in the mouse models. The group showed that at the early stage, heterozygous Tbk1 deletion impairs autophagy in motoneurons and prepones the clinical onset and muscular denervation in SOD1G93A/Tbk1 ± mice, while at the late disease stage, it significantly alleviates microglial neuroinflammation, decelerates disease progression, and extends mouse survival (Brenner et al., 2019).

### Summary

After several decades of research, it is now clear that the same genes can cause ALS and FTD. Mutations in C9orf72, TARDBP, FUS, TBK1, VCP, CHCHD10, and SQSTM1 are the most closely associated with both diseases. Clinically, the ALS phenotype is most commonly associated with the behavioral variant of FTD, with other subtypes of FTD involving language occurring less commonly. The pathophysiology underlying this observation is poorly understood.

Nevertheless, this overlap is not complete: SOD1, FUS, and TDP-43 variants are most commonly associated with ALS and are only rarely found in FTD patients. Similarly, GRN is linked to FTD, but not to ALS. Clinically, the ALS phenotype is most commonly associated with the behavioral variant of FTD, with other subtypes of FTD involving language occurring less commonly. The pathophysiology underlying this observation is poorly understood.

It is striking how the same pathways are implicated repeatedly in ALS and FTD. Both disorders characterized by defects in RNA processing, protein clearance by autophagy, vesicle trafficking, mitochondrial dysfunction, and impaired protein homeostasis. The genes described in this review are the key players in these pathways. TDP-43 and FUS are responsible for RNA regulation; SQSTM1, C9orf72, VCP, and TBK1 are involved in autophagy and vesicle dynamics; TDP-43, FUS, and SQSTM1 are common components of nuclear and cytoplasmic inclusions (Weishaupt et al., 2016). Due to such significant genetic overlap between ALS and FTD, it is reasonable to look in FTD cases for mutations in ALS genes, and vice-versa.

The C9orf72 repeat expansion gives rise to a diverse range of inter-familial and intra-familial phenotypes, including age at disease onset, site of symptom onset, rate and pattern of progression, levels of cognitive impairment and motor neuron degeneration, as well as disease duration. This clinical heterogeneity likely indicates that both genetic and environmental factors play a significant role in the development and course of the disease. Environmental factors such as occupational exposure to heavy metals, toxic compounds, and extremely low-frequency electromagnetic frequencies have been previously reported to increase the risk of developing neurological disorders. Studies on personal habits revealed an increased risk of ALS among smokers, as well as an overall worse prognosis after disease onset. In contrast, alcohol consumption was associated with a reduced risk of ALS. Literature analysis of head trauma and the development of neurological disorders were inconclusive. More recently, advanced genetic analysis of a large genetic dataset implicated high cholesterol as driving the risk of ALS, as well as confirming an association with smoking and physical exercise (Bandres-Ciga et al., 2019).

Research shows that environmental factors can influence people’s chances of developing ALS or FTD. Nevertheless, the studies were performed on case cohorts that were not genetically selected. Different sets of environmental factors may interact with different genes. Consequently, future genetic epidemiology efforts should focus on cohorts selected based on their underlying genetic risk. Studying such population-based cohorts that have been assiduously collected and phenotyped for clinical features, genetics, epigenetics, and environmental and lifestyle exposures will be essential to these efforts.

## Author Contributions

YA drafted the manuscript. PF, RC, and BT participated in critically revising the manuscript for important intellectual content. All authors read and approved the final manuscript.

## Conflict of Interest

BT has a European patent granted and US patent pending on the clinical testing and therapeutic intervention for the hexanucleotide repeat expansion of C9orf72. The remaining authors declare that the research was conducted in the absence of any commercial or financial relationships that could be construed as a potential conflict of interest. The handling Editor declared a past co-authorship with one of the authors BT. The reviewer PM declared a past co-authorship with one of the authors BT to the handling Editor.

## References

[B1] Ajroud-Driss S. Fecto F. Ajroud K. Lalani I. Calvo S. E. Mootha V. K. (2015). Mutation in the novel nuclear-encoded mitochondrial protein CHCHD10 in a family with autosomal dominant mitochondrial myopathy. *Neurogenetics* 16 1–9. 10.1007/s10048-014-0421-1 25193783 PMC4796476

[B2] Al-Obeidi E. Al-Tahan S. Surampalli A. Goyal N. Wang A. K. Hermann A. (2018). Genotype-phenotype study in patients with valosin-containing protein mutations associated with multisystem proteinopathy. *Clin. Genet.* 93 119–125. 10.1111/cge.13095 28692196 PMC5739971

[B3] An H. Skelt L. Notaro A. Highley J. R. Fox A. H. La Bella V. (2019). ALS-linked FUS mutations confer loss and gain of function in the nucleus by promoting excessive formation of dysfunctional paraspeckles. *Acta Neuropathol. Commun.* 7:7. 10.1186/s40478-019-0658-x 30642400 PMC6330737

[B4] Anderson C. J. Bredvik K. Burstein S. R. Davis C. Meadows S. M. Dash J. (2019). ALS/FTD mutant CHCHD10 mice reveal a tissue-specific toxic gain-of-function and mitochondrial stress response. *Acta Neuropathol.* 138 103–121. 10.1007/s00401-019-01989-y 30877432 PMC6571048

[B5] Arai T. Nonaka T. Hasegawa M. Akiyama H. Yoshida M. Hashizume Y. (2003). Neuronal and glial inclusions in frontotemporal dementia with or without motor neuron disease are immunopositive for p62. *Neurosci. Lett.* 342 41–44. 12727313 10.1016/s0304-3940(03)00216-7

[B6] Arhzaouy K. Papadopoulos C. Schulze N. Pittman S. K. Meyer H. Weihl C. C. (2019). VCP maintains lysosomal homeostasis and TFEB activity in differentiated skeletal muscle. *Autophagy* 15 1082–1099. 10.1080/15548627.2019.1569933 30654731 PMC6526815

[B7] Armstrong R. A. (2017). Neuronal cytoplasmic inclusions in tau, TDP-43, and FUS molecular subtypes of frontotemporal lobar degeneration share similar spatial patterns. *Folia Neuropathol.* 55 185–192. 10.5114/fn.2017.70482 28984110

[B8] Arthur K. C. Calvo A. Price T. R. Geiger J. T. Chio A. Traynor B. J. (2016). Projected increase in amyotrophic lateral sclerosis from 2015 to 2040. *Nat. Commun.* 7:12408. 10.1038/ncomms12408 27510634 PMC4987527

[B9] Arzberger T. Schludi M. H. Lehmer C. Schmid B. Edbauer D. (2018). RNA versus protein toxicity in C9orf72 ALS/FTLD. *Acta Neuropathol.* 135 475–479. 29450647 10.1007/s00401-018-1823-1

[B10] Bandres-Ciga S. Noyce A. J. Hemani G. Nicolas A. Calvo A. Mora G. (2019). Shared polygenic risk and causal inferences in amyotrophic lateral sclerosis. *Ann. Neurol.* 85 470–481. 10.1002/ana.25431 30723964 PMC6450729

[B11] Bannwarth S. Ait-El-Mkadem S. Chaussenot A. Genin E. C. Lacas-Gervais S. Fragaki K. (2014). A mitochondrial origin for frontotemporal dementia and amyotrophic lateral sclerosis through CHCHD10 involvement. *Brain* 137(Pt 8), 2329–2345. 10.1093/brain/awu138 24934289 PMC4107737

[B12] Bastola P. Bilkis R. De Souza C. Minn K. Chien J. (2019). Heterozygous mutations in valosin-containing protein (VCP) and resistance to VCP inhibitors. *Sci. Rep.* 9:11002. 10.1038/s41598-019-47085-9 31358864 PMC6662852

[B13] Beck J. Poulter M. Hensman D. Rohrer J. D. Mahoney C. J. Adamson G. (2013). Large C9orf72 hexanucleotide repeat expansions are seen in multiple neurodegenerative syndromes and are more frequent than expected in the UK population. *Am. J. Hum. Genet.* 92 345–353. 10.1016/j.ajhg.2013.01.011 23434116 PMC3591848

[B14] Berry J. Brangwynne C. P. Haataja M. (2018). Physical principles of intracellular organization via active and passive phase transitions. *Rep. Prog. Phys.* 81:046601. 10.1088/1361-6633/aaa61e 29313527

[B15] Bird T. D. Nochlin D. Poorkaj P. Cherrier M. Kaye J. Payami H. (1999). A clinical pathological comparison of three families with frontotemporal dementia and identical mutations in the tau gene (P301L). *Brain* 122(Pt 4), 741–756. 10219785 10.1093/brain/122.4.741

[B16] Bodansky A. Kim J. M. Tempest L. Velagapudi A. Libby R. Ravits J. (2010). TDP-43 and ubiquitinated cytoplasmic aggregates in sporadic ALS are low frequency and widely distributed in the lower motor neuron columns independent of disease spread. *Amyotroph. Lateral. Scler.* 11 321–327. 10.3109/17482961003602363 20225928 PMC4981144

[B17] Bonnard M. Mirtsos C. Suzuki S. Graham K. Huang J. Ng M. (2000). Deficiency of T2K leads to apoptotic liver degeneration and impaired NF-kappaB-dependent gene transcription. *EMBO J.* 19 4976–4985. 10990461 10.1093/emboj/19.18.4976 PMC314216

[B18] Brenner D. Sieverding K. Bruno C. Luningschror P. Buck E. Mungwa S. (2019). Heterozygous Tbk1 loss has opposing effects in early and late stages of ALS in mice. *J. Exp. Med.* 216 267–278. 10.1084/jem.20180729 30635357 PMC6363427

[B19] Buratti E. Baralle F. E. (2008). Multiple roles of TDP-43 in gene expression, splicing regulation, and human disease. *Front Biosci.* 13:867–878. 17981595 10.2741/2727

[B20] Buratti E. Baralle F. E. (2012). TDP-43: gumming up neurons through protein-protein and protein-RNA interactions. *Trends Biochem. Sci.* 37 237–247. 10.1016/j.tibs.2012.03.003 22534659

[B21] Buratti E. Dork T. Zuccato E. Pagani F. Romano M. Baralle F. E. (2001). Nuclear factor TDP-43 and SR proteins promote in vitro and in vivo CFTR exon 9 skipping. *EMBO J.* 20 1774–1784. 11285240 10.1093/emboj/20.7.1774 PMC145463

[B22] Burrell J. R. Kiernan M. C. Vucic S. Hodges J. R. (2011). Motor neuron dysfunction in frontotemporal dementia. *Brain* 134(Pt 9), 2582–2594. 10.1093/brain/awr195 21840887

[B23] Burstein S. R. Valsecchi F. Kawamata H. Bourens M. Zeng R. Zuberi A. (2018). In vitro and in vivo studies of the ALS-FTLD protein CHCHD10 reveal novel mitochondrial topology and protein interactions. *Hum. Mol. Genet.* 27 160–177. 10.1093/hmg/ddx397 29112723 PMC5886281

[B24] Chaussenot A. Le Ber I. Ait-El-Mkadem S. Camuzat A. de Septenville A. Bannwarth S. (2014). Screening of CHCHD10 in a French cohort confirms the involvement of this gene in frontotemporal dementia with amyotrophic lateral sclerosis patients. *Neurobiol. Aging* 35 e1–e4. 10.1016/j.neurobiolaging.2014.07.022 25155093

[B25] Chio A. Logroscino G. Traynor B. J. Collins J. Simeone J. C. Goldstein L. A. (2013). Global epidemiology of amyotrophic lateral sclerosis: a systematic review of the published literature. *Neuroepidemiology* 41 118–130. 10.1159/000351153 23860588 PMC4049265

[B26] Choi S. Y. Lopez-Gonzalez R. Krishnan G. Phillips H. L. Li A. N. Seeley W. W. (2019). C9ORF72-ALS/FTD-associated poly(GR) binds Atp5a1 and compromises mitochondrial function in vivo. *Nat. Neurosci.* 22 851–862. 10.1038/s41593-019-0397-0 31086314 PMC6800116

[B27] Cirulli E. T. Lasseigne B. N. Petrovski S. Sapp P. C. Dion P. A. Leblond C. S. (2015). Exome sequencing in amyotrophic lateral sclerosis identifies risk genes and pathways. *Science* 347 1436–1441. 10.1126/science.aaa3650 25700176 PMC4437632

[B28] Cleveland D. W. Rothstein J. D. (2001). From Charcot to Lou Gehrig: deciphering selective motor neuron death in ALS. *Nat. Rev. Neurosci.* 2 806–819. 11715057 10.1038/35097565

[B29] Conicella A. E. Zerze G. H. Mittal J. Fawzi N. L. (2016). ALS mutations disrupt phase separation mediated by alpha-helical structure in the TDP-43 low-complexity C-terminal domain. *Structure* 24 1537–1549. 10.1016/j.str.2016.07.007 27545621 PMC5014597

[B30] Cozzolino M. Rossi S. Mirra A. Carri M. T. (2015). Mitochondrial dynamism and the pathogenesis of Amyotrophic Lateral Sclerosis. *Front. Cell Neurosci.* 9:31. 10.3389/fncel.2015.00031 25713513 PMC4322717

[B31] Davidson Y. Kelley T. Mackenzie I. R. Pickering-Brown S. Du Plessis D. Neary D. (2007). Ubiquitinated pathological lesions in frontotemporal lobar degeneration contain the TAR DNA-binding protein. TDP-43. *Acta Neuropathol.* 113 521–533. 17219193 10.1007/s00401-006-0189-y

[B32] de Majo M. Topp S. D. Smith B. N. Nishimura A. L. Chen H. J. Gkazi A. S. (2018). ALS-associated missense and nonsense TBK1 mutations can both cause loss of kinase function. *Neurobiol. Aging* 71 e1–e10. 10.1016/j.neurobiolaging.2018.06.015 30033073 PMC6983933

[B33] DeJesus-Hernandez M. Mackenzie I. R. Boeve B. F. Boxer A. L. Baker M. Rutherford N. J. (2011). Expanded GGGGCC hexanucleotide repeat in noncoding region of C9ORF72 causes chromosome 9p-linked FTD and ALS. *Neuron* 2011 245–256. 10.1016/j.neuron.2011.09.011 21944778 PMC3202986

[B34] Deng Z. Lim J. Wang Q. Purtell K. Wu S. Palomo G. M. (2019). ALS-FTLD-linked mutations of SQSTM1/p62 disrupt selective autophagy and NFE2L2/NRF2 anti-oxidative stress pathway. *Autophagy* 10.1080/15548627.2019.1644076 [Epub ahead of print], 31362587 PMC7144840

[B35] Devenney E. M. Ahmed R. M. Halliday G. Piguet O. Kiernan M. C. Hodges J. R. (2018). Psychiatric disorders in C9orf72 kindreds: study of 1,414 family members. *Neurology* 91 e1498–e1507. 10.1212/WNL.0000000000006344 30258023

[B36] Devoy A. Kalmar B. Stewart M. Park H. Burke B. Noy S. J. (2017). Humanized mutant FUS drives progressive motor neuron degeneration without aggregation in ‘FUSDelta14’ knockin mice. *Brain* 140 2797–2805. 10.1093/brain/awx248 29053787 PMC5841203

[B37] Duan W. Guo M. Yi L. Zhang J. Bi Y. Liu Y. (2019). Deletion of Tbk1 disrupts autophagy and reproduces behavioral and locomotor symptoms of FTD-ALS in mice. *Aging* 11 2457–2476. 10.18632/aging.101936 31039129 PMC6519994

[B38] Ebstein S. Y. Yagudayeva I. Shneider N. A. (2019). Mutant TDP-43 causes early-stage dose-dependent motor neuron degeneration in a tardbp knockin mouse model of ALS. *Cell Rep* 26:364-373e4. 10.1016/j.celrep.2018.12.045 30625319

[B39] Fecto F. Yan J. Vemula S. P. Liu E. Yang Y. Chen W. (2011). SQSTM1 mutations in familial and sporadic amyotrophic lateral sclerosis. *Arch Neurol.* 68 1440–1446. 10.1001/archneurol.2011.250 22084127

[B40] Fiesel F. C. Voigt A. Weber S. S. Van den Haute C. Waldenmaier A. Gorner K. (2010). Knockdown of transactive response DNA-binding protein (TDP-43) downregulates histone deacetylase 6. *EMBO J.* 29 209–221. 10.1038/emboj.2009.324 19910924 PMC2808372

[B41] Foster A. Scott D. Layfield R. Rea S. L. (2019). An FTLD-associated SQSTM1 variant impacts Nrf2 and NF-kappaB signalling and is associated with reduced phosphorylation of p62. *Mol. Cell Neurosci.* 98 32–45. 10.1016/j.mcn.2019.04.001 30954537

[B42] Fratta P. Sivakumar P. Humphrey J. Lo K. Ricketts T. Oliveira H. (2018). Mice with endogenous TDP-43 mutations exhibit gain of splicing function and characteristics of amyotrophic lateral sclerosis. *EMBO J.* 37:e98684. 10.15252/embj.201798684 29764981 PMC5983119

[B43] Freibaum B. D. Lu Y. Lopez-Gonzalez R. Kim N. C. Almeida S. Lee K. H. (2015). GGGGCC repeat expansion in C9orf72 compromises nucleocytoplasmic transport. *Nature* 525 129–133. 10.1038/nature14974 26308899 PMC4631399

[B44] Freibaum B. D. Taylor J. P. (2017). The role of dipeptide repeats in C9ORF72-related ALS-FTD. *Front. Mol. Neurosci.* 10:35. 10.3389/fnmol.2017.00035 28243191 PMC5303742

[B45] Freischmidt A. Wieland T. Richter B. Ruf W. Schaeffer V. Muller K. (2015). Haploinsufficiency of TBK1 causes familial ALS and fronto-temporal dementia. *Nat. Neurosci.* 18 631–636. 10.1038/nn.4000 25803835

[B46] Ganji R. Mukkavalli S. Somanji F. Raman M. (2018). The VCP-UBXN1 complex mediates triage of ubiquitylated cytosolic proteins bound to the BAG6 complex. *Mol. Cell Biol.* 38:MCB.00154-118. 10.1128/MCB.00154-18 29685906 PMC6002697

[B47] Gao J. Perera G. Bhadbhade M. Halliday G. M. Dzamko N. (2019). Autophagy activation promotes clearance of alpha-synuclein inclusions in fibril-seeded human neural cells. *J. Biol. Chem.* 294 14241–14256. 10.1074/jbc.RA119.008733 31375560 PMC6768637

[B48] Genin E. C. Madji Hounoum B. Bannwarth S. Fragaki K. Lacas-Gervais S. Mauri-Crouzet A. (2019). Mitochondrial defect in muscle precedes neuromuscular junction degeneration and motor neuron death in CHCHD10(S59L/+) mouse. *Acta Neuropathol.* 138 123–145. 10.1007/s00401-019-01988-z 30874923

[B49] Hao Z. Liu L. Tao Z. Wang R. Ren H. Sun H. (2019). Motor dysfunction and neurodegeneration in a C9orf72 mouse line expressing poly-PR. *Nat. Commun.* 10:2906. 10.1038/s41467-019-10956-w 31266945 PMC6606620

[B50] Hensman Moss D. J. Poulter M. Beck J. Hehir J. Polke J. M. Campbell T. (2014). C9orf72 expansions are the most common genetic cause of Huntington disease phenocopies. *Neurology* 82 292–299. 10.1212/WNL.0000000000000061 24363131 PMC3929197

[B51] Hergesheimer R. C. Chami A. A. de Assis D. R. Vourc’h P. Andres C. R. Corcia P. (2019). The debated toxic role of aggregated TDP-43 in amyotrophic lateral sclerosis: a resolution in sight? *Brain* 142 1176–1194. 10.1093/brain/awz078 30938443 PMC6487324

[B52] Herhaus L. Dikic I. (2018). Ubiquitin-induced phase separation of p62/SQSTM1. *Cell Res.* 28 389–390. 29572488 10.1038/s41422-018-0030-x PMC5939040

[B53] Ho W. Y. Ling S. C. (2019). Elevated FUS levels by overriding its autoregulation produce gain-of-toxicity properties that disrupt protein and RNA homeostasis. *Autophagy* 15 1665–1667. 10.1080/15548627.2019.1633162 31230528 PMC6693452

[B54] Hu Y. W. Zhang J. Wu X. M. Cao L. Nie P. Chang M. X. (2018). TANK-binding kinase 1 (TBK1) isoforms negatively regulate type I Interferon Induction by inhibiting TBK1-IRF3 interaction and IRF3 phosphorylation. *Front. Immunol.* 9:84. 10.3389/fimmu.2018.00084 29441066 PMC5797597

[B55] Huey E. D. Ferrari R. Moreno J. H. Jensen C. Morris C. M. Potocnik F. (2012). FUS and TDP43 genetic variability in FTD and CBS. *Neurobiol. Aging* 33 e9–e17. 10.1016/j.neurobiolaging.2011.08.004 21943958 PMC4489700

[B56] Ishigaki S. Sobue G. (2018). Importance of functional loss of FUS in FTLD/ALS. *Front. Mol. Biosci.* 5:44. 10.3389/fmolb.2018.00044 29774215 PMC5943504

[B57] Jiang J. Zhu Q. Gendron T. F. Saberi S. McAlonis-Downes M. Seelman A. (2016). Gain of toxicity from ALS/FTD-linked repeat expansions in C9ORF72 Is alleviated by antisense oligonucleotides targeting GGGGCC-containing RNAs. *Neuron* 90 535–550. 10.1016/j.neuron.2016.04.006 27112497 PMC4860075

[B58] Johnson J. O. Mandrioli J. Benatar M. Abramzon Y. Van Deerlin V. M. Trojanowski J. Q. (2010). Exome sequencing reveals VCP mutations as a cause of familial ALS. *Neuron* 68 857–864. 10.1016/j.neuron.2010.11.036 21145000 PMC3032425

[B59] Jovicic A. Mertens J. Boeynaems S. Bogaert E. Chai N. Yamada S. B. (2015). Modifiers of C9orf72 dipeptide repeat toxicity connect nucleocytoplasmic transport defects to FTD/ALS. *Nat. Neurosci.* 18 1226–1229. 10.1038/nn.4085 26308983 PMC4552077

[B60] Jung K. T. Oh S. H. (2019). Polyubiquitination of p62/SQSTM1 is a prerequisite for Fas/CD95 aggregation to promote caspase-dependent apoptosis in cadmium-exposed mouse monocyte RAW264.7 cells. *Sci. Rep.* 9:12240. 10.1038/s41598-019-48684-2 31439879 PMC6706394

[B61] Kang J. Lim L. Lu Y. Song J. (2019). A unified mechanism for LLPS of ALS/FTLD-causing FUS as well as its modulation by ATP and oligonucleic acids. *PLoS Biol.* 17:e3000327. 10.1371/journal.pbio.3000327 31188823 PMC6590835

[B62] Khalfallah Y. Kuta R. Grasmuck C. Prat A. Durham H. D. Vande Velde C. (2018). TDP-43 regulation of stress granule dynamics in neurodegenerative disease-relevant cell types. *Sci. Rep.* 8:7551. 10.1038/s41598-018-25767-0 29765078 PMC5953947

[B63] Kino Y. Washizu C. Kurosawa M. Yamada M. Miyazaki H. Akagi T. (2015). FUS/TLS deficiency causes behavioral and pathological abnormalities distinct from amyotrophic lateral sclerosis. *Acta Neuropathol. Commun.* 3:24. 10.1186/s40478-015-0202-6 25907258 PMC4408580

[B64] Koppers M. Blokhuis A. M. Westeneng H. J. Terpstra M. L. Zundel C. A. Vieira de Sa R. (2015). C9orf72 ablation in mice does not cause motor neuron degeneration or motor deficits. *Ann. Neurol.* 78 426–438. 10.1002/ana.24453 26044557 PMC4744979

[B65] Kwiatkowski T. J. Jr. Bosco D. A. Leclerc A. L. Tamrazian E. Vanderburg C. R. Russ C. (2009). Mutations in the FUS/TLS gene on chromosome 16 cause familial amyotrophic lateral sclerosis. *Science* 323 1205–1208. 10.1126/science.1166066 19251627

[B66] Kwon J. Han E. Bui C. B. Shin W. Lee J. Lee S. (2012). Assurance of mitochondrial integrity and mammalian longevity by the p62-Keap1-Nrf2-Nqo1 cascade. *EMBO Rep.* 13 150–156. 10.1038/embor.2011.246 22222206 PMC3271336

[B67] Lagier-Tourenne C. Polymenidou M. Cleveland D. W. (2010). TDP-43 and FUS/TLS: emerging roles in RNA processing and neurodegeneration. *Hum. Mol. Genet.* 19 R46–R64. 10.1093/hmg/ddq137 20400460 PMC3167692

[B68] Lamb R. Rohrer J. D. Real R. Lubbe S. J. Waite A. J. Blake D. J. (2019). A novel TBK1 mutation in a family with diverse frontotemporal dementia spectrum disorders. *Cold Spring Harb. Mol. Case Stud.* 5:a003913. 10.1101/mcs.a003913 31160356 PMC6549548

[B69] Laurin N. Brown J. P. Morissette J. Raymond V. (2002). Recurrent mutation of the gene encoding sequestosome 1 (SQSTM1/p62) in Paget disease of bone. *Am. J. Hum. Genet.* 70 1582–1588. 11992264 10.1086/340731 PMC379146

[B70] Li F. Xie X. Wang Y. Liu J. Cheng X. Guo Y. (2016). Structural insights into the interaction and disease mechanism of neurodegenerative disease-associated optineurin and TBK1 proteins. *Nat. Commun.* 7:12708. 10.1038/ncomms12708 27620379 PMC5027247

[B71] Ling S. C. Dastidar S. G. Tokunaga S. Ho W. Y. Lim K. Ilieva H. (2019). Overriding FUS autoregulation in mice triggers gain-of-toxic dysfunctions in RNA metabolism and autophagy-lysosome axis. *eLife* 8:e40811. 10.7554/eLife.40811 30747709 PMC6389288

[B72] Ludtmann M. H. Arber C. Bartolome F. de Vicente M. Preza E. Carro E. (2017). Mutations in valosin-containing protein (VCP) decrease ADP/ATP translocation across the mitochondrial membrane and impair energy metabolism in human neurons. *J. Biol. Chem.* 292 8907–8917. 10.1074/jbc.M116.762898 28360103 PMC5448124

[B73] Mackenzie I. R. Rademakers R. Neumann M. (2010). TDP-43 and FUS in amyotrophic lateral sclerosis and frontotemporal dementia. *Lancet Neurol.* 9 995–1007. 10.1016/S1474-4422(10)70195-2 20864052

[B74] May S. Hornburg D. Schludi M. H. Arzberger T. Rentzsch K. Schwenk B. M. (2014). C9orf72 FTLD/ALS-associated Gly-Ala dipeptide repeat proteins cause neuronal toxicity and Unc119 sequestration. *Acta Neuropathol.* 128 485–503. 10.1007/s00401-014-1329-4 25120191 PMC4159571

[B75] Miller R. G. Mitchell J. D. Moore D. H. (2012). Riluzole for amyotrophic lateral sclerosis (ALS)/motor neuron disease (MND). *Cochrane Database Syst. Rev.* 3 CD001447. 10.1002/14651858.CD001447 11687111

[B76] Mitra J. Guerrero E. N. Hegde P. M. Liachko N. F. Wang H. Vasquez V. (2019). Motor neuron disease-associated loss of nuclear TDP-43 is linked to DNA double-strand break repair defects. *Proc. Natl. Acad. Sci. U.S.A.* 116 4696–4705. 10.1073/pnas.1818415116 30770445 PMC6410842

[B77] Mitsui S. Otomo A. Nozaki M. Ono S. Sato K. Shirakawa R. (2018). Systemic overexpression of SQSTM1/p62 accelerates disease onset in a SOD1(H46R)-expressing ALS mouse model. *Mol. Brain* 11:30. 10.1186/s13041-018-0373-8 29843805 PMC5975400

[B78] Morton S. Hesson L. Peggie M. Cohen P. (2008). Enhanced binding of TBK1 by an optineurin mutant that causes a familial form of primary open angle glaucoma. *FEBS Lett.* 582 997–1002. 10.1016/j.febslet.2008.02.047 18307994

[B79] Murthy A. C. Dignon G. L. Kan Y. Zerze G. H. Parekh S. H. Mittal J. (2019). Molecular interactions underlying liquid-liquid phase separation of the FUS low-complexity domain. *Nat. Struct. Mol. Biol.* 26 637–648. 10.1038/s41594-019-0250-x 31270472 PMC6613800

[B80] Nalbandian A. Khan A. A. Srivastava R. Llewellyn K. J. Tan B. Shukr N. (2017). Activation of the NLRP3 inflammasome is associated with valosin-containing protein myopathy. *Inflammation* 40 21–41. 10.1007/s10753-016-0449-5 27730320 PMC5800525

[B81] Neumann M. Sampathu D. M. Kwong L. K. Truax A. C. Micsenyi M. C. Chou T. T. (2006). Ubiquitinated TDP-43 in frontotemporal lobar degeneration and amyotrophic lateral sclerosis. *Science* 314 130–133. 17023659 10.1126/science.1134108

[B82] Niaki A. G. Sarkar J. Cai X. Rhine K. Vidaurre V. Guy B. (2019). Loss of dynamic RNA interaction and aberrant phase separation induced by two distinct types of ALS/FTD-Linked FUS mutations. *Mol. Cell* 77:82-94.e4. 10.1016/j.molcel.2019.09.022 31630970 PMC6943187

[B83] Ogura T. Wilkinson A. J. (2001). AAA+ superfamily ATPases: common structure–diverse function. *Genes Cells* 6 575–597. 11473577 10.1046/j.1365-2443.2001.00447.x

[B84] O’Rourke J. G. Bogdanik L. Muhammad A. K. Gendron T. F. Kim K. J. Austin A. (2015). C9orf72 BAC transgenic mice display typical pathologic features of ALS/FTD. *Neuron* 88 892–901. 10.1016/j.neuron.2015.10.027 26637796 PMC4672384

[B85] Park S. Zuber C. Roth J. (2019). Selective autophagy of cytosolic protein aggregates involves ribosome-free rough endoplasmic reticulum. *Histochem. Cell Biol.* 10.1007/s00418-019-01829-w [Epub ahead of print], 31720797

[B86] Perrone F. Nguyen H. P. Van Mossevelde S. Moisse M. Sieben A. Santens P. (2017). Investigating the role of ALS genes CHCHD10 and TUBA4A in Belgian FTD-ALS spectrum patients. *Neurobiol. Aging.* 51 e9–e16. 10.1016/j.neurobiolaging.2016.12.008 28069311

[B87] Perry A. K. Chow E. K. Goodnough J. B. Yeh W. C. Cheng G. (2004). Differential requirement for TANK-binding kinase-1 in type I interferon responses to toll-like receptor activation and viral infection. *J. Exp. Med.* 199 1651–1658. 15210743 10.1084/jem.20040528 PMC2212814

[B88] Peters O. M. Cabrera G. T. Tran H. Gendron T. F. McKeon J. E. Metterville J. (2015). Human C9ORF72 hexanucleotide expansion reproduces RNA Foci and dipeptide repeat proteins but not neurodegeneration in BAC transgenic mice. *Neuron* 88 902–909. 10.1016/j.neuron.2015.11.018 26637797 PMC4828340

[B89] Plewa J. Surampalli A. Wencel M. Milad M. Donkervoort S. Caiozzo V. J. (2018). A cross-sectional analysis of clinical evaluation in 35 individuals with mutations of the valosin-containing protein gene. *Neuromuscul. Disord.* 28 778–786. 10.1016/j.nmd.2018.06.007 30097247 PMC6490182

[B90] Ratnavalli E. Brayne C. Dawson K. Hodges J. R. (2002). The prevalence of frontotemporal dementia. *Neurology* 58 1615–1621. 12058088 10.1212/wnl.58.11.1615

[B91] Rea S. L. Walsh J. P. Layfield R. Ratajczak T. Xu J. (2013). New insights into the role of sequestosome 1/p62 mutant proteins in the pathogenesis of Paget’s disease of bone. *Endocr. Rev.* 34 501–524. 10.1210/er.2012-1034 23612225

[B92] Renton A. E. Majounie E. Waite A. Simon-Sanchez J. Rollinson S. Gibbs J. R. (2011). A hexanucleotide repeat expansion in C9ORF72 is the cause of chromosome 9p21-linked ALS-FTD. *Neuron* 72 257–268. 10.1016/j.neuron.2011.09.010 21944779 PMC3200438

[B93] Roczniak-Ferguson A. Ferguson S. M. (2019). Pleiotropic requirements for human TDP-43 in the regulation of cell and organelle homeostasis. *bioRxiv [Preprint]* 10.26508/lsa.20190035 31527135 PMC6749094

[B94] Rothstein J. D. (2017). Edaravone: a new drug approved for ALS. *Cell* 171:725. 10.1016/j.cell.2017.10.011 29100067

[B95] Ryzhakov G. Randow F. (2007). SINTBAD, a novel component of innate antiviral immunity, shares a TBK1-binding domain with NAP1 and TANK. *EMBO J.* 26 3180–3190. 17568778 10.1038/sj.emboj.7601743 PMC1914091

[B96] Saracino D. Clot F. Camuzat A. Anquetil V. Hannequin D. Guyant-Marechal L. (2018). Novel VCP mutations expand the mutational spectrum of frontotemporal dementia. *Neurobiol. Aging* 72 e11–e14. 10.1016/j.neurobiolaging.2018.06.037 30005904

[B97] Scekic-Zahirovic J. Sendscheid O. El Oussini H. Jambeau M. Sun Y. Mersmann S. (2016). Toxic gain of function from mutant FUS protein is crucial to trigger cell autonomous motor neuron loss. *EMBO J.* 35 1077–1097. 10.15252/embj.201592559 26951610 PMC4868956

[B98] Shang Y. Huang E. J. (2016). Mechanisms of FUS mutations in familial amyotrophic lateral sclerosis. *Brain Res.* 1647 65–78. 10.1016/j.brainres.2016.03.036 27033831 PMC5003642

[B99] Shao Q. Liang C. Chang Q. Zhang W. Yang M. Chen J. F. (2019). C9orf72 deficiency promotes motor deficits of a C9ALS/FTD mouse model in a dose-dependent manner. *Acta Neuropathol. Commun.* 7:32. 10.1186/s40478-019-0685-7 PMC6398253 30832726

[B100] Shi Y. Lin S. Staats K. A. Li Y. Chang W. H. Hung S. T. (2018). Haploinsufficiency leads to neurodegeneration in C9ORF72 ALS/FTD human induced motor neurons. *Nat. Med.* 24 313–325. 10.1038/nm.4490 29400714 PMC6112156

[B101] Silva P. R. Nieva G. V. Igaz L. M. (2019). Suppression of conditional TDP-43 transgene expression differentially affects early cognitive and social phenotypes in TDP-43 Mice. *Front. Genet.* 10:369. 10.3389/fgene.2019.00369 31068973 PMC6491777

[B102] Sreedharan J. Blair I. P. Tripathi V. B. Hu X. Vance C. Rogelj B. (2008). TDP-43 mutations in familial and sporadic amyotrophic lateral sclerosis. *Science* 319 1668–1672. 10.1126/science.1154584 18309045 PMC7116650

[B103] Strong M. J. Volkening K. Hammond R. Yang W. Strong W. Leystra-Lantz C. (2007). TDP43 is a human low molecular weight neurofilament (hNFL) mRNA-binding protein. *Mol. Cell Neurosci.* 35 320–327. 17481916 10.1016/j.mcn.2007.03.007

[B104] Sun D. Wu R. Zheng J. Li P. Yu L. (2018). Polyubiquitin chain-induced p62 phase separation drives autophagic cargo segregation. *Cell Res.* 28 405–415. 10.1038/s41422-018-0017-7 29507397 PMC5939046

[B105] Tan R. H. Yang Y. Kim W. S. Dobson-Stone C. Kwok J. B. Kiernan M. C. (2017). Distinct TDP-43 inclusion morphologies in frontotemporal lobar degeneration with and without amyotrophic lateral sclerosis. *Acta Neuropathol. Commun.* 5:76. 10.1186/s40478-017-0480-2 29078806 PMC5658959

[B106] Taylor J. P. Brown R. H. Jr. Cleveland D. W. (2016). Decoding ALS: from genes to mechanism. *Nature* 539 197–206. 10.1038/nature20413 27830784 PMC5585017

[B107] Teyssou E. Takeda T. Lebon V. Boillee S. Doukoure B. Bataillon G. (2013). Mutations in SQSTM1 encoding p62 in amyotrophic lateral sclerosis: genetics and neuropathology. *Acta Neuropathol.* 125 511–522. 23417734 10.1007/s00401-013-1090-0

[B108] Tsai R. M. Boxer A. L. (2014). Treatment of frontotemporal dementia. *Curr. Treat. Options Neurol.* 16:319. 10.1007/s11940-014-0319-0 25238733 PMC4920050

[B109] Twomey E. C. Ji Z. Wales T. E. Bodnar N. O. Ficarro S. B. Marto J. A. (2019). Substrate processing by the Cdc48 ATPase complex is initiated by ubiquitin unfolding. *Science* 365:1033. 10.1126/science.aax1033 31249135 PMC6980381

[B110] Uhlen M. Fagerberg L. Hallstrom B. M. Lindskog C. Oksvold P. Mardinoglu A. (2015). Proteomics. Tissue-based map of the human proteome. *Science* 347:1260419. 10.1126/science.1260419 25613900

[B111] Van Langenhove T. van der Zee J. Sleegers K. Engelborghs S. Vandenberghe R. Gijselinck I. (2010). Genetic contribution of FUS to frontotemporal lobar degeneration. *Neurology* 74 366–371. 10.1212/WNL.0b013e3181ccc732 20124201

[B112] Van Mossevelde S. van der Zee J. Gijselinck I. Engelborghs S. Sieben A. Van Langenhove T. (2016). Clinical features of TBK1 carriers compared with C9orf72, GRN and non-mutation carriers in a Belgian cohort. *Brain* 139(Pt 2), 452–467. 10.1093/brain/awv358 26674655 PMC4805085

[B113] van Rheenen W. Shatunov A. Dekker A. M. McLaughlin R. L. Diekstra F. P. Pulit S. L. (2016). Genome-wide association analyses identify new risk variants and the genetic architecture of amyotrophic lateral sclerosis. *Nat. Genet.* 48 1043–1048. 10.1038/ng.3622 27455348 PMC5556360

[B114] Vance C. Rogelj B. Hortobagyi T. De Vos K. J. Nishimura A. L. Sreedharan J. (2009). Mutations in FUS, an RNA processing protein, cause familial amyotrophic lateral sclerosis type 6. *Science* 323 1208–1211. 10.1126/science.1165942 19251628 PMC4516382

[B115] Wang R. Yang B. Zhang D. (2011). Activation of interferon signaling pathways in spinal cord astrocytes from an ALS mouse model. *Glia* 59 946–958. 10.1002/glia.21167 21446050 PMC3077460

[B116] Watts G. D. Wymer J. Kovach M. J. Mehta S. G. Mumm S. Darvish D. (2004). Inclusion body myopathy associated with Paget disease of bone and frontotemporal dementia is caused by mutant valosin-containing protein. *Nat. Genet.* 36 377–381. 15034582 10.1038/ng1332

[B117] Weidberg H. Elazar Z. (2011). TBK1 mediates crosstalk between the innate immune response and autophagy. *Sci. Signal.* 4:e39. 10.1126/scisignal.2002355 21868362

[B118] Weinreich M. Shepheard S. R. Verber N. Wyles M. Heath P. R. Highley J. R. (2019). Neuropathological characterization of a novel TANK binding kinase (TBK1) gene loss of function mutation associated with amyotrophic lateral sclerosis. *Neuropathol. Appl. Neurobiol.* 10.1111/nan.12578 [Epub ahead of print]. 31498468

[B119] Weishaupt J. H. Hyman T. Dikic I. (2016). Common molecular pathways in amyotrophic lateral sclerosis and frontotemporal dementia. *Trends Mol. Med.* 22 769–783. 10.1016/j.molmed.2016.07.005 27498188

[B120] White M. A. Kim E. Duffy A. Adalbert R. Phillips B. U. Peters O. M. (2018a). Publisher Correction: TDP-43 gains function due to perturbed autoregulation in a Tardbp knock-in mouse model of ALS-FTD. *Nat. Neurosci.* 21:1138. 10.1038/s41593-018-0160-y 29872124

[B121] White M. A. Kim E. Duffy A. Adalbert R. Phillips B. U. Peters O. M. (2018b). TDP-43 gains function due to perturbed autoregulation in a Tardbp knock-in mouse model of ALS-FTD. *Nat. Neurosci.* 21 552–563. 29556029 10.1038/s41593-018-0113-5 PMC5884423

[B122] Xiao Y. Zhang J. Shu X. Bai L. Xu W. Wang A. (2019). Loss of mitochondrial protein CHCHD10 in skeletal muscle causes neuromuscular junction impairment. *Hum. Mol. Genet.* 10.1093/hmg/ddz154 [Epub ahead of print]. 31261376

[B123] Yang Y. Willis T. L. Button R. W. Strang C. J. Fu Y. Wen X. (2019). Cytoplasmic DAXX drives SQSTM1/p62 phase condensation to activate Nrf2-mediated stress response. *Nat. Commun.* 10:3759. 10.1038/s41467-019-11671-2 31434890 PMC6704147

[B124] Zaffagnini G. Savova A. Danieli A. Romanov J. Tremel S. Ebner M. (2018). Phasing out the bad-How SQSTM1/p62 sequesters ubiquitinated proteins for degradation by autophagy. *Autophagy* 14 1280–1282. 10.1080/15548627.2018.1462079 29929426 PMC6103668

[B125] Zhang K. Donnelly C. J. Haeusler A. R. Grima J. C. Machamer J. B. Steinwald P. (2015). The C9orf72 repeat expansion disrupts nucleocytoplasmic transport. *Nature* 525 56–61. 10.1038/nature14973 26308891 PMC4800742

[B126] Zhang Y. J. Gendron T. F. Ebbert M. T. W. O’Raw A. D. Yue M. Jansen-West K. (2018). Poly(GR) impairs protein translation and stress granule dynamics in C9orf72-associated frontotemporal dementia and amyotrophic lateral sclerosis. *Nat. Med.* 24 1136–1142. 10.1038/s41591-018-0071-1 29942091 PMC6520050

[B127] Zhou Z. D. Saw W. T. Tan E. K. (2017). Mitochondrial CHCHD-containing proteins: physiologic functions and link with neurodegenerative diseases. *Mol. Neurobiol.* 54 5534–5546. 10.1007/s12035-016-0099-5 27631878

